# The top 100 cited articles on artificial intelligence in Alzheimer’s disease and mild cognitive impairment: a bibliometric analysis

**DOI:** 10.3389/fnagi.2025.1605231

**Published:** 2026-01-12

**Authors:** Zhi Tao, Rui Zhou, Yinggang Zheng, Lize Xiong

**Affiliations:** Shanghai Key Laboratory of Anesthesiology and Brain Functional Modulation, Department of Anesthesiology and Perioperative Medicine, Clinical Research Center for Anesthesiology and Perioperative Medicine, Translational Research Institute of Brain and Brain-Like Intelligence, Shanghai Fourth People’s Hospital, School of Medicine, Tongji University, Shanghai, China

**Keywords:** Alzheimer’s disease, artificial intelligence, bibliometric analysis, machine learning, mild cognitive impairment, top 100

## Abstract

**Background:**

Alzheimer’s disease (AD) and Mild Cognitive Impairment (MCI) pose significant societal and healthcare burden. Artificial intelligence (AI) methods have been widely applied in AD and MCI studies. We conducted a bibliometric analysis of the 100 most cited articles on AI applied to AD and MCI.

**Methods:**

We searched the Web of Science database using keywords related to AD, MCI, and AI (e.g., “deep learning,” “machine learning,” “neural networks”). Citation counts ranked articles, and the top 100 were manually screened. Key parameters such as authors, journals, citation count, countries, institutions, and keywords were automatically extracted. We also manually extracted key information, including publication type, impact factor (IF), Journal Citation Reports (JCR) Category Quartile, AI methods, and clinical data types. Analysis and visualization were conducted using VOSviewer.

**Results:**

Among the 100 articles, 13 were reviews, 2 were basic research papers, and 85 were clinical studies. Seventy seven articles focused on diagnosis and prediction. MRI data was the most frequently used analysis source. Shen Dinggang, the United States, and the University of North Carolina at Chapel Hill were respectively the individual, country, and institution with the highest publication volume. Neuroimage published the most papers (*n* = 14), and all the top 10 journals belonged to JCR Q1. Emerging keywords included “ensemble learning,” “transfer learning,” and “structural MRI.” Support Vector Machine (SVM) was the most commonly applied AI method (*n* = 25), closely followed by convolutional neural network (CNN, *n* = 24).

**Conclusion:**

We analyzed the top 100 cited articles on AI in AD and MCI across authors, journals, countries, institutions, keywords, and AI methods. Diagnosing AD/MCI is the primary research focus, with MRI as the most studied examination. SVM and CNN are the most frequently used AI methods in these studies.

## 1 Introduction

Currently, more than 55 million people worldwide are living with dementia, the seventh leading cause of death and one of the primary causes of disability and dependency among older adults (World Health Organization [WHO], 2024). Alzheimer’s disease (AD), a serious brain disorder caused by damage to neurons, is the main cause of dementia (Alzheimers Dementia, 2024; Taylor et al., 2017; Petersen et al., 2018; Rabinovici et al., 2019). As the global population ages, the number of individuals affected by AD is expected to rise. In 2020, it was estimated that 6.07 million older adults in the United States had clinical AD and this number was projected to increase by 18% to 7.16 million by 2025 and 128% to 13.85 million by 2060 (Rajan et al., 2021). Such an increase will impose a heavy financial burden on the society. The estimated global cost of caring for individuals with dementia is expected to rise to approximately $2 trillion by 2030 (World Health Organization [WHO], 2024).

Traditional clinical medications for AD, such as donepezil, rivastigmine, galantamine, and memantine, may cause side effects like arrhythmia, dizziness, headache, and nausea, and they do not alter the disease’s progression (Nham et al., 2024; Henstra et al., 2022; Maelicke et al., 2010; Tan et al., 2014). The efficacy of non-pharmacological interventions, such as music and reminiscence therapy, remains uncertain. New drugs like lecanemab aim to change the underlying biology of AD by targeting and slowing cognitive and functional decline. However, lecanemab is only suitable for patients with early AD, and the safety and efficacy of long-term use have not been observed yet (Terao and Kodama, 2024, 2024; van Dyck et al., 2023). Thus, it is particularly important to diagnose AD as early as possible. Mild cognitive impairment (MCI) represents a cognitive function state that lies between normal aging and dementia. Studies have shown that patients with MCI have a significantly increased risk of progressing to AD compared with the general population (Petersen et al., 2018; Rabinovici et al., 2019).

Many methods have been applied to the auxiliary diagnosis of AD, including the Clinical Dementia Rating Scale (CDR), magnetic resonance imaging (MRI), cerebrospinal fluid (CSF) biomarkers, and positron emission tomography (PET). However, there is still a high misdiagnosis rate in the early diagnosis of dementia in clinical practice (Beach et al., 2012; Gianattasio et al., 2019; Hunter et al., 2015; Nguyen et al., 2022). The underdiagnosis of MCI is even more pronounced, with only 8% of older adults with MCI in the United States receiving a diagnosis (Liu et al., 2024).

Artificial intelligence (AI) is the simulation of human intelligence in machines to make them think and learn like humans. It includes a variety of types, such as machine learning (ML), deep learning (DL), computer vision, and expert systems. AI has found widespread applications in medicine. For example, the application of AI in imaging diagnosis has progressed notably in the identification of tumors, such as prostate and breast cancer (Jiang et al., 2024; Molière et al., 2024). Furthermore, a great number of studies have been conducted in the realm of cognitive function diagnostics (Hussain and Borah, 2024; Basaia et al., 2019; Myszczynska et al., 2020). We conducted a literature search in the Web of Science (WoS) Core Collection and got 22,646 records, highlighting the vibrancy of research in this field.

Highly cited articles can serve as important indicators of the research status and trends within a specific field, providing valuable insights that can significantly enhance clinical practice. To effectively summarize key information and assist future researchers in gaining a clearer understanding of the characteristics of published works in this domain, we intend to analyze the top 100 cited articles on the combination of AI and AD/MCI using bibliometric methods.

## 2 Materials and methods

### 2.1 Database and retrieval tactics

WoS, a widely utilized database for evaluating scientific research outputs, quality, and developmental trends, was selected as the data source for this study. The search strategy was as follows: TS = (“machine learning” OR “ML” OR “deep learning” OR “neural network” OR “neural networks” OR “artificial intelligence” OR “AI” OR “predictive modeling” OR “predictive analytics”) AND TS = (“Mild Cognitive Impairment” OR “MCI” OR “Alzheimer’s disease” OR “Alzheimer diseases” OR “AD”). Our search was conducted within the WoS Core Collection. The article types were restricted to Article and Review Article. No language restrictions were applied in the search process. Data acquisition occurred on November 4, 2024.

### 2.2 Data collection and analysis

Studies on the application of AI or ML to AD and/or MCI were eligible. We included “Article” and “Review Article” in the analysis. Other types, such as “Letter” and “Proceedings Letter” were not included. Given that some classic articles may have been published years ago, we did not impose any restrictions on the publication year; all available records were considered. The observed concentration of highly cited papers between 2013 and 2019 reflects natural citation accumulation patterns rather than a predefined time limit. First, we ranked the retrieved articles in descending order of citation count. The top 100 cited articles were then manually screened for analysis. We automatically extracted 29 uncensored bibliometric parameters from the WoS Core Collection. The data for the top 100 cited articles included the authors, article title, source title (journal name), publication year, keywords, institutions, countries/regions, and citation count. Subsequently, two researchers identified and extracted the publication type, impact factor (IF), Journal Citation Reports (JCR) Category Quartile, ML methods, and clinical data types analyzed from the journals. Finally, any discrepancies between the two researchers were reviewed and resolved through discussion. We then used a professional tool VOSviewer (version 1.6.20) for basic analysis and visualization.

## 3 Results

### 3.1 General analysis

**FIGURE 1:**
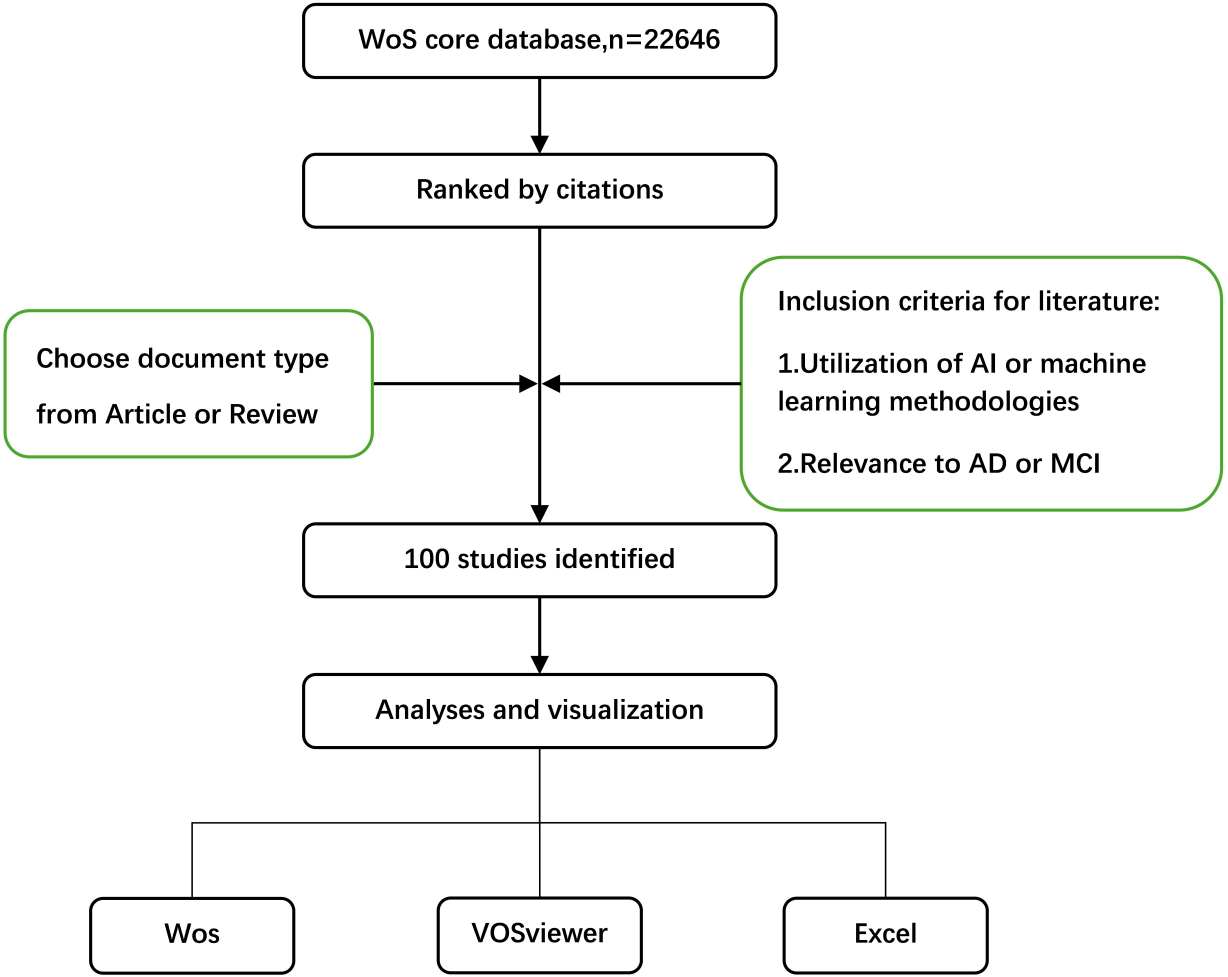
Flowchart of the study. Web of Science (WoS).

The flowchart detailing the methodology of this study is presented in Figure 1.

**FIGURE 2:**
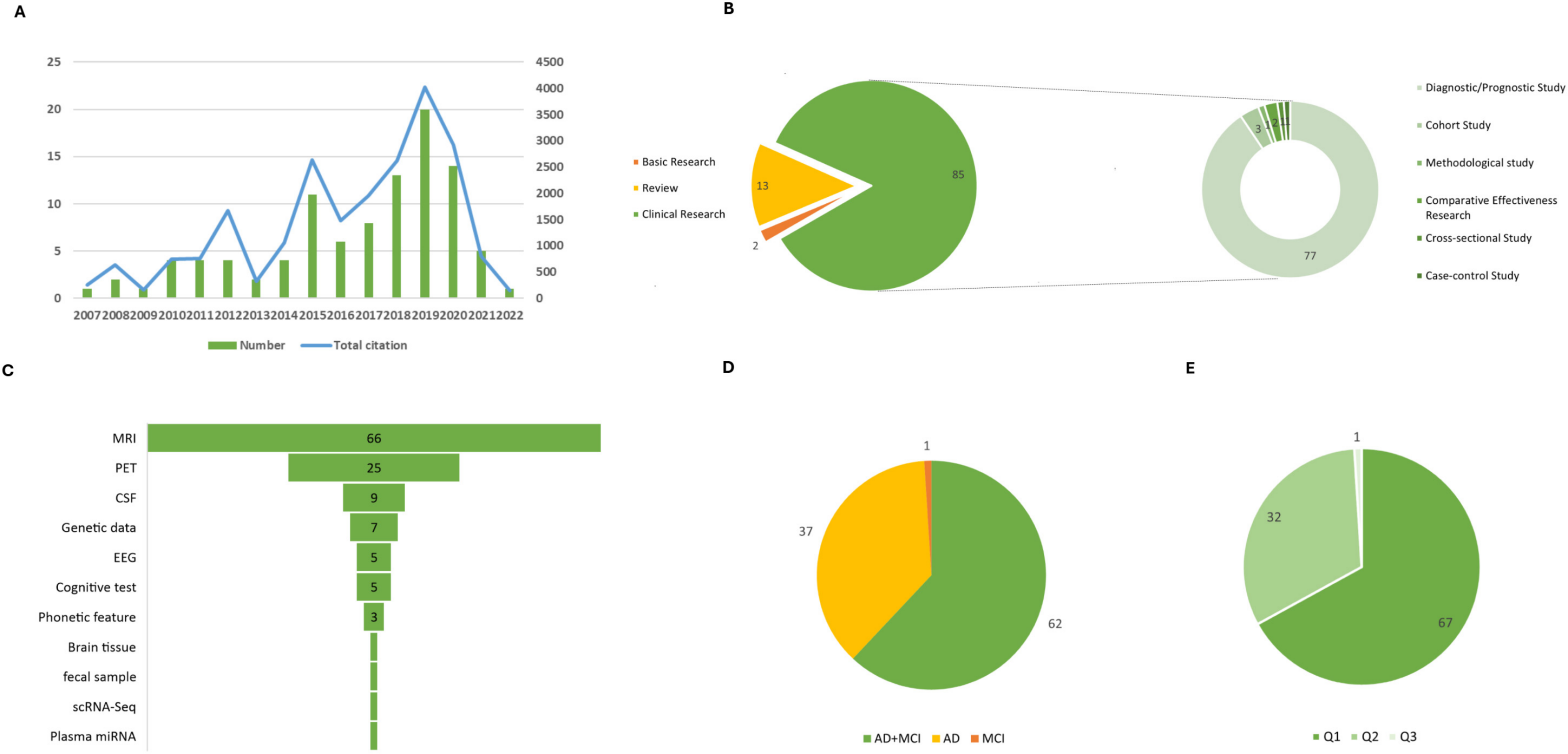
General analysis. **(A)** Distribution of the 100 articles by publication year. **(B)** Proportions of different study designs. **(C)** Types of clinical data source. **(D)** Proportions of studies associated with AD, MCI, and both AD and MCI. **(E)** Proportions by JCR category quartile.

As shown in Figure 2A, the publication years of the top 100 cited papers ranged from 2007 to 2022, with a sharp growth after 2013 and a peak between 2018 and 2019. This pattern indicates that the high-impact studies mainly emerged during the rapid expansion of AI applications in AD/MCI research, rather than reflecting any pre-set time limitation of our analysis. In Figure 2B, among these 100 articles, there were 13 review articles, 2 basic studies, and 85 clinical studies. Within clinical research, diagnosis and prediction studies were the main types, encompassing 77 articles.

The primary types of clinical specimens collected in clinical research include MRI, PET-CT scans, CSF, electroencephalogram (EEG), genetic data, phonetic features, cognitive tests, brain tissue samples, plasma miRNA, fecal samples, and single-cell RNA sequencing (scRNA-Seq). The statistical results are displayed in Figure 2C.

We further classified and analyzed the literature, as shown in Figure 2D. Specifically, 67 articles primarily examined both AD and MCI, 32 articles focused solely on AD, and 1 article concentrated on MCI exclusively. Notably, MRI emerged as the most frequently utilized imaging modality, serving as a critical variable in 66 studies for ML analysis. This was followed by PET, which appeared in 25 articles, CSF findings in 9 articles, and genetic data featured in seven articles.

Of the articles reviewed, 68 were classified within the JCR Q1 zone, 31 in the JCR Q2 zone, and 1 in the JCR Q3 zone, as shown in Figure 2E.

### 3.2 Author analyses

**FIGURE 3:**
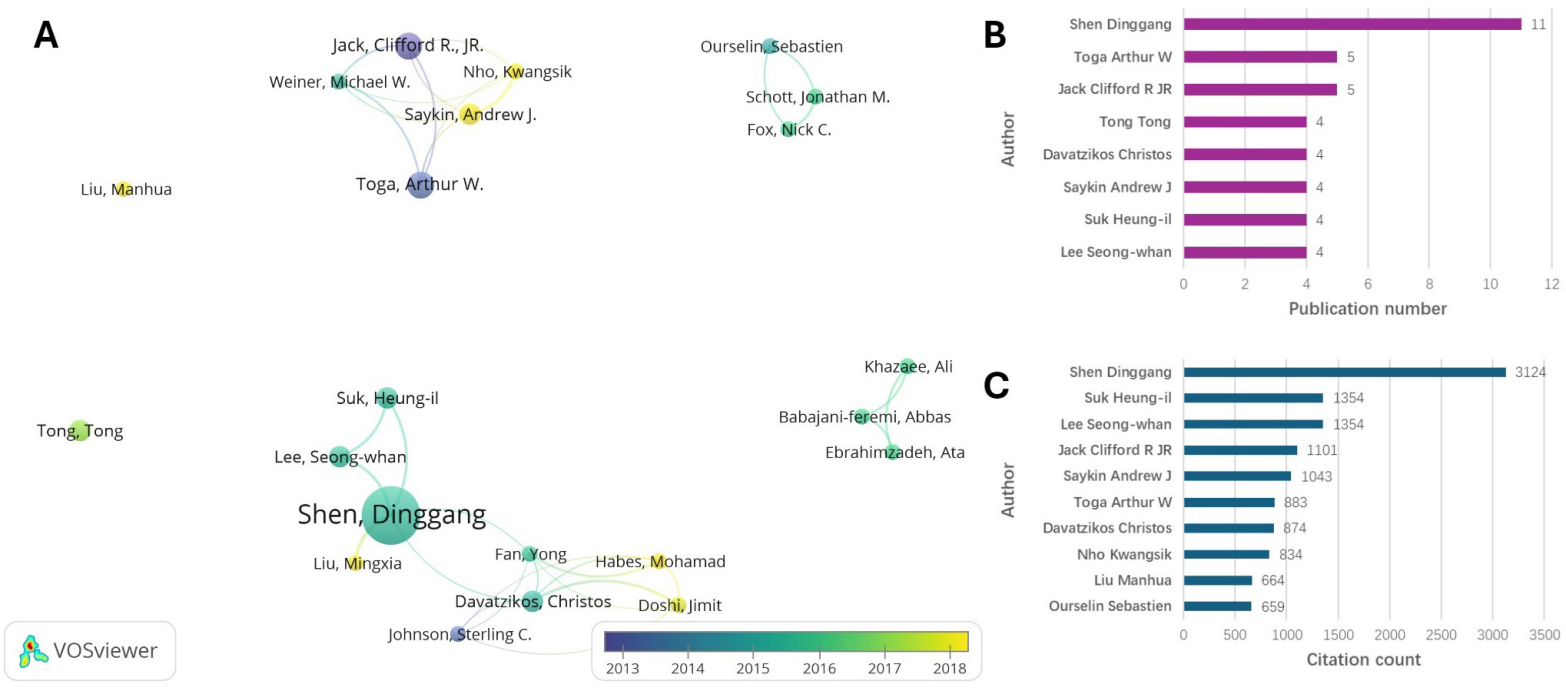
Author analyses. **(A)** Visualized connections of the authors. **(B)** Top eight authors of publication number. **(C)** Top 10 authors of citation count.

There were 979 authors among the top 100 relevant articles, with 22 authors having published three or more articles. To better understand the relationships among these authors, we utilized VOSviewer to analyze the strength of their connections based on mutual citations. Shen Dinggang demonstrated the highest level of interaction with other authors (Figure 3A). Additionally, Figure 3A also depicts the timeline of the author’s publications. Doshi Jimit, Habes Mohamad, Saykin Andrew J., Nho Kwangsik, Liu Mingxia, and Liu Manhua primarily published their papers after 2018. Figure 3B illustrates the top eight authors ranked by publication number, while Figure 3C presents the top 10 authors based on total citations. Specifically, Shen Dinggang was identified as the most prolific author, with a total of 11 publications, followed by Toga Arthur W. and Jack Clifford R. JR (five articles). Shen Dinggang also had the most citations. Several emerging authors including Liu Mingxia and Liu Manhua, who mostly published after 2018, appeared among the top 100 cited articles despite having fewer total citations compared to established authors like Shen Dinggang. This indicates rapid progress from newer research teams in recent years.

### 3.3 Analyses of country/region and institution

**FIGURE 4:**
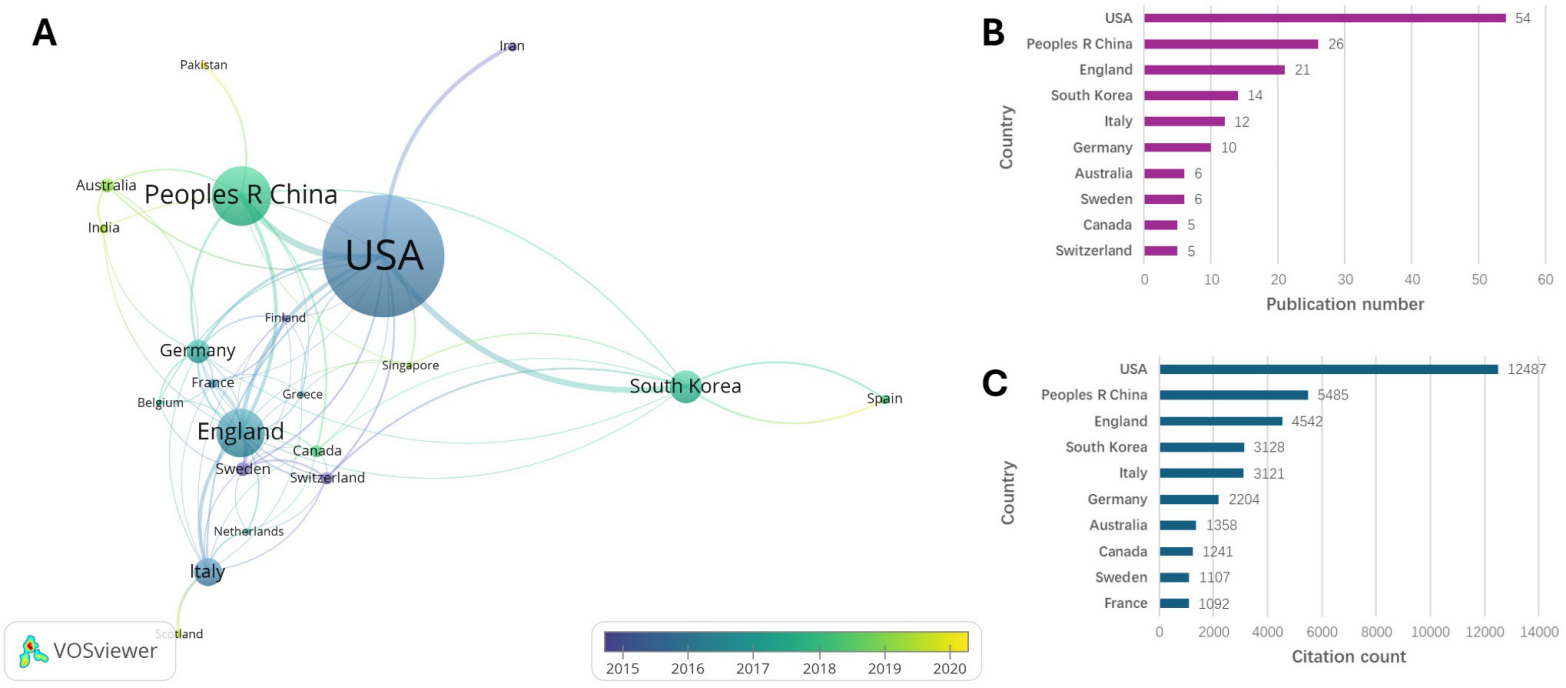
Country analyses. **(A)** Visualized connections between the countries/regions. **(B)** Top 10 countries/regions of publication number. **(C)** Top 10 countries/regions of citation count.

**FIGURE 5:**
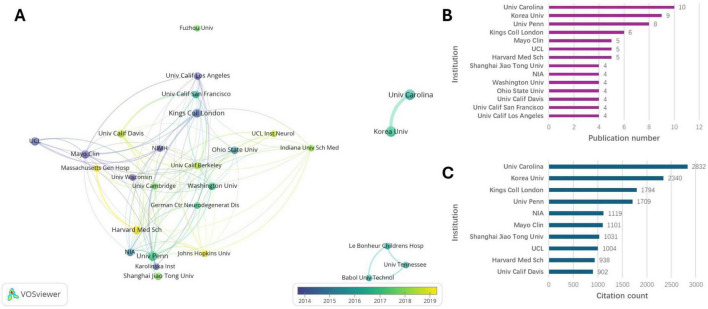
Institution analyses. **(A)** Visualized connections between the Institutions. **(B)** Top 13 institutions of publication number. **(C)** Top 10 Institutions of citation count.

Figure 4A illustrates the collaboration network among countries/regions. As shown in Figures 4B,C, the United States had the highest number of publications and citations, with 54 publications accounting for more than half of the total. China ranked second with 26 articles, followed by England with 14, South Korea with 14, and Italy with 12. Figure 5A indicates a strong collaborative relationship between the University of North Carolina at Chapel Hill and Korea University. Among the top 14 institutions, 10 are located in the United States (Figure 5B), including the University of North Carolina at Chapel Hill, the University of Pennsylvania, Harvard Medical School, the Mayo Clinic, the University of California Los Angeles, the University of California San Francisco, the University of California Davis, the Ohio State University, the University of Washington, and the National Institute on Aging (NIA). Among the 10 most productive institutions highlighted in Figures 5B,C, the University of North Carolina at Chapel Hill emerged as the institution with the highest number of publications and citations. Besides the United States and China, countries like England, South Korea, and Italy contributed several papers to the top 100 despite lower citation totals. Korea University and University College London published highly cited papers post-2018, showing their growing role in the field.

### 3.4 Journal analyses

**FIGURE 6:**
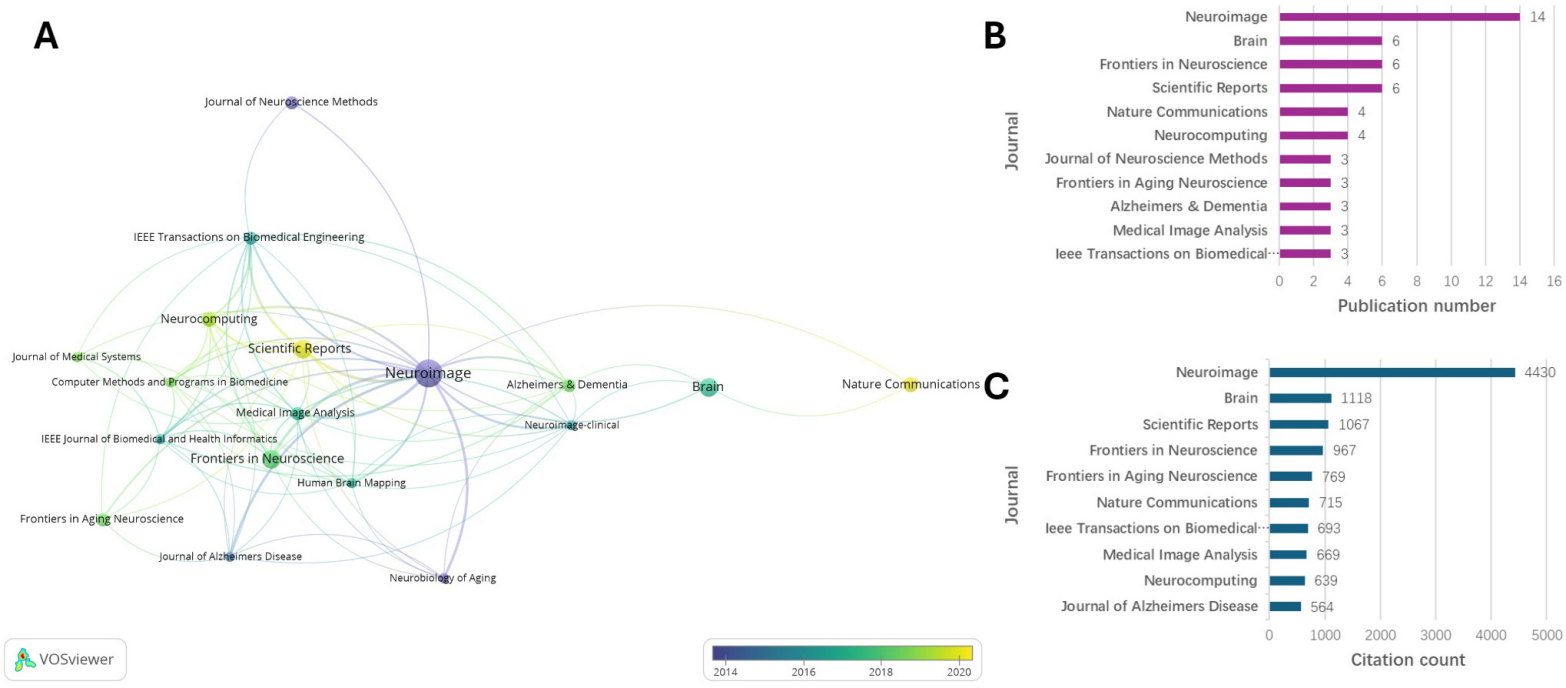
Journal analyses. **(A)** Visualized connections between the journals. **(B)** Top 11 journals of publication number. **(C)** Top 10 journals of citation count.

The analyzed papers were published in 48 different journals, with 19 of those journals publishing more than two articles. We conducted a cluster analysis of these journals, revealing that Nature Communications and Scientific Reports had the most recent publications, primarily around 2020 (Figure 6A). Neuroimage was both the most frequently published and the most cited journal, contributing a total of 14 relevant articles as shown in Figures 6B,C. Brain, Frontiers in Neuroscience, and Scientific Reports each published six papers, while Nature Communications and Neurocomputing each published four papers. Table 1 presents the top 10 journals with the highest impact factors, along with their respective impact factors, number of publications, JCR quartiles, and JCR categories. Notably, the top 10 journals are classified within the JCR Q1 region.

**TABLE 1:** The 10 journals with the highest impact factor (IF).

Rank	Journal	IF_2023_	Publications	JCR quartile	JCR category
1	Nature Reviews Neurology	28.2	1	Q1	Clinical neurology
2	Nature Biomedical Engineering	27.7	1	Q1	Engineering, biomedical
3	IEEE Transactions on Pattern Analysis and Machine Intelligence	20.8	1	Q1	Computer science, artificial intelligence; engineering, electrical and electronic
4	Nature Communications	14.7	4	Q1	Multidisciplinary sciences
5	Alzheimer’s and Dementia	13.1	3	Q1	Clinical neurology
6	Radiology	12.1	1	Q1	Radiology, nuclear medicine and medical imaging
7	Brain	11.9	6	Q1	Clinical neurology; neurosciences
8	Medical Image Analysis	10.7	3	Q1	Radiology, nuclear medicine, and medical imaging; computer science, interdisciplinary applications; computer science, artificial intelligence; engineering, biomedical
9	PLoS Medicine	10.5	1	Q1	Medicine, general, and internal
10	IEEE Transactions on Medical Imaging	8.9	1	Q1	Imaging science, and photographic technology; radiology, nuclear medicine and medical imaging; computer science, interdisciplinary applications; engineering, electrical and electronic; engineering, biomedical

Q1: the first quartile. JCR, journal citation reports.

### 3.5 Keywords analyses

**FIGURE 7:**
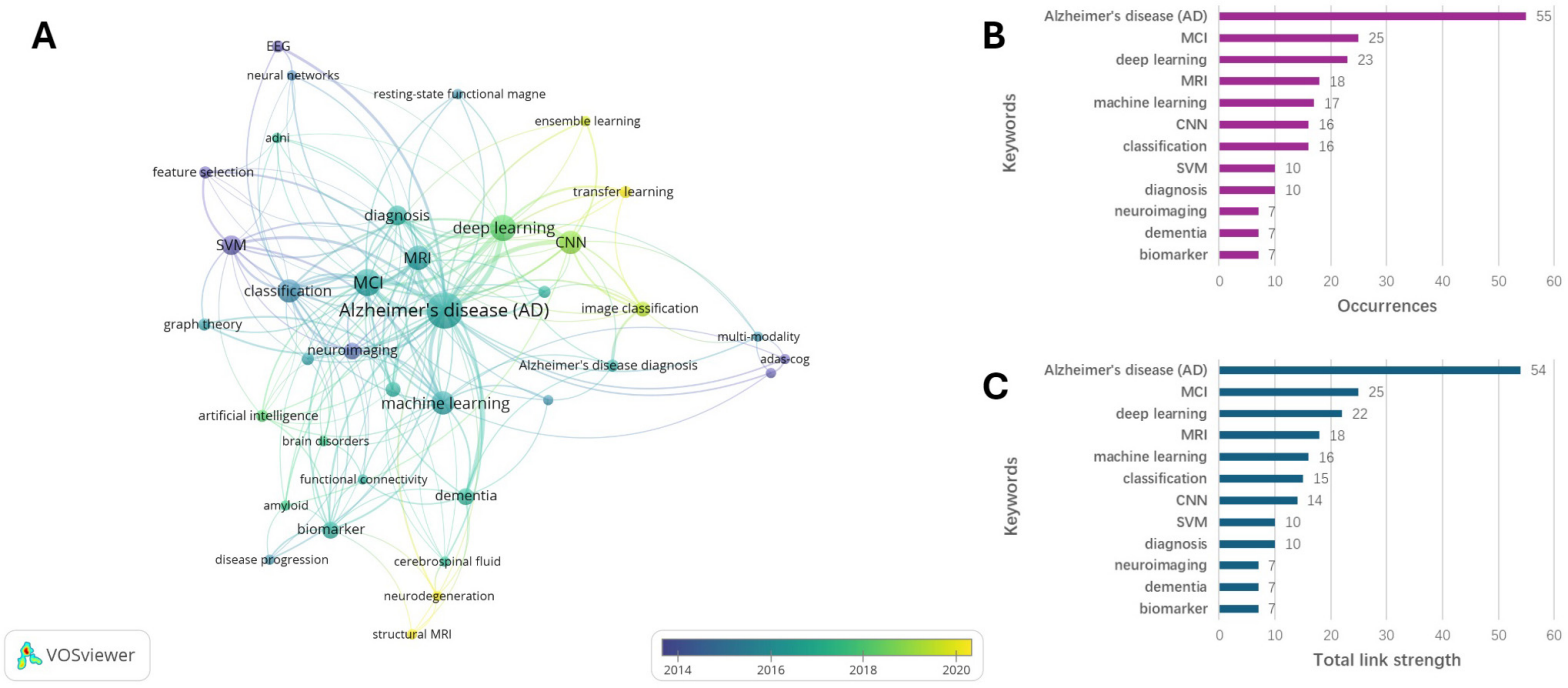
Keywords analyses. **(A)** Visualized connections between the keywords. **(B)** Top 12 keywords of publication number. **(C)** Top 12 keywords of citation count.

We performed a co-occurrence analysis of all keywords using VOSviewer. As shown in Figure 7A, keywords such as “ensemble learning,” “transfer learning,” “neurodegeneration,” and “structural MRI” have begun to emerge over time, indicating potential new directions for future research. Figure 7B displays the top 12 keywords, with “Alzheimer’s disease” appearing most frequently (55 times), followed by “MCI” (25 times), and “deep learning” (23 times). Other notable keywords include “MRI” (18 times), “machine learning” (17 times), “convolutional neural network” (CNN) (16 times), and “classification” (16 times). The results of the keywords ranked by total link strength and occurrences were largely consistent (Figures 7B,C). In addition, the emergence of “ensemble learning” and “transfer learning” in recent years indicates a shift in methodological focus. Earlier studies were mainly associated with keywords such as “SVM” and “CNN,” whereas the newer terms suggest a move toward approaches that emphasize model integration, robustness, and improved generalizability in heterogeneous or limited datasets.

### 3.6 Analyses of AI methods

**FIGURE 8:**
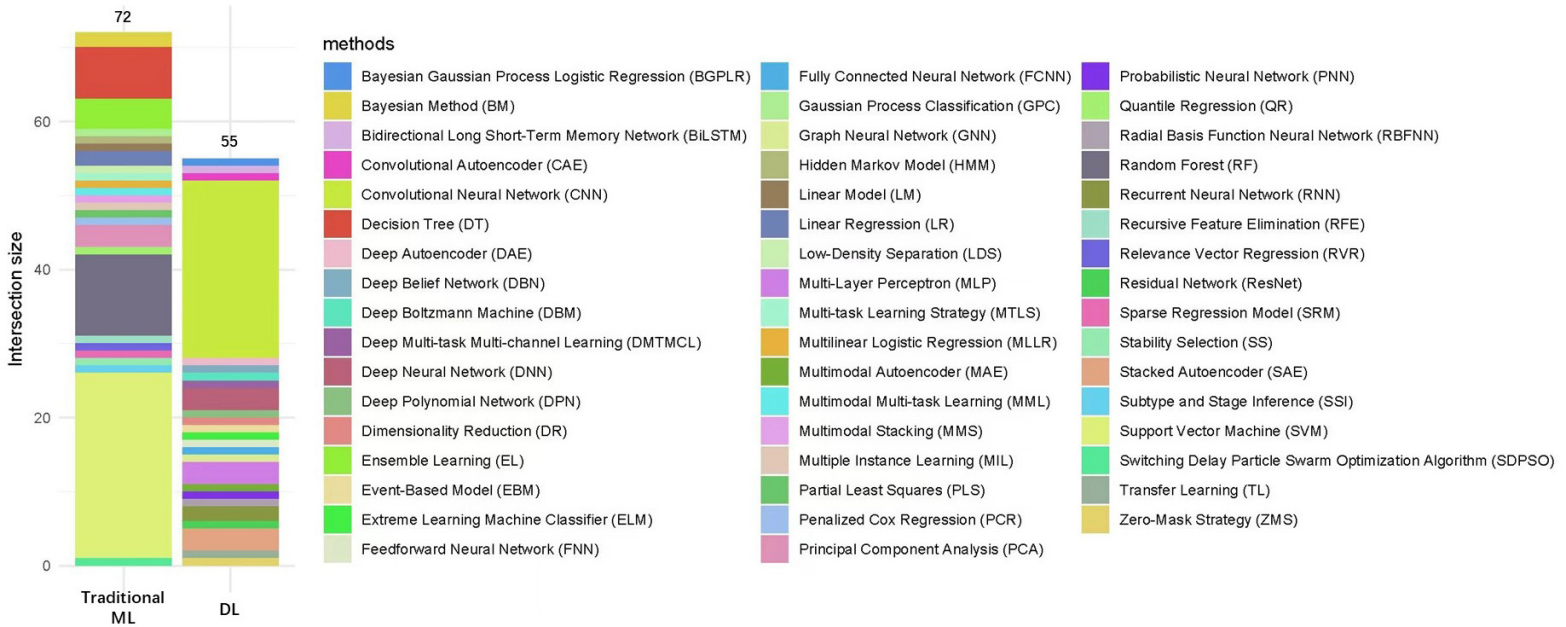
Frequency of the 50 specific AI methods. This figure presents a statistical analysis of 50 distinct AI methods. The methods are categorized into two main groups: Traditional Machine Learning (ML) and Deep Learning (DL), all categorized under AI. Traditional ML (left side) refers to ML methods that do not involve DL techniques. These methods rely on statistical and algorithmic models. DL involves neural networks and complex models, which learn from large, high-dimensional datasets. The frequency of each method’s appearance across the articles is represented by bar lengths, with each bar color corresponding to a specific method listed on the right. The size of each bar represents the intersection size, i.e., the number of articles in which the method was applied. Traditional ML methods were utilized 72 times. DL methods were used 55 times.

We conducted a detailed statistical analysis of 50 AI methods used across the 87 research articles focusing on AD/MCI. Figure 8 represents the frequency of these methods. Among these methods, those classified solely as traditional ML (excluding DL) were utilized 72 times, while DL methods were used 55 times. The figure also shows a wide range of methods with various frequencies, reflecting the diverse approaches that have been explored in AD/MCI research. The 12 most commonly used methods are displayed in Table 2. Notably, the Support Vector Machine (SVM) was the most frequently applied method, appearing in 25 articles. This was followed by the CNN, cited in 24 articles, the Random Forest (RF) method, used in 11 articles, the Decision Tree (DT) method, employed in seven articles, and Ensemble Learning (EL), appeared in four articles.

**TABLE 2:** Twelve AI methods ranked by occurrence.

Methods	Count	Classification
Support vector machine (SVM)	25	Traditional ML
Convolutional neural network (CNN)	24	DL
Random forest (RF)	11	Traditional ML
Decision tree (DT)	7	Traditional ML
Ensemble learning (EL)	4	Traditional ML
Stacked autoencoder (SAE)	3	DL
Principal component analysis (PCA)	3	Traditional ML
Multi-layer perceptron (MLP)	3	DL
Deep neural network (DNN)	3	DL
Linear regression (LR)	2	Traditional ML
Recurrent neural network (RNN)	2	DL
Bayesian method (BM)	2	Traditional ML

## 4 Discussion

We conducted a bibliometric analysis of the top 100 most cited articles on AI in AD/MCI. The main findings include: (1) diagnostic and predictive studies are the most frequent research types, with MRI being the most commonly used clinical data source; (2) SVM and CNN are the most commonly applied AI methods; (3) the United States and China are the leading countries contributing to AI-driven AD/MCI research; (4) high-impact journals such as Neuroimage, Frontiers in Neuroscience, and Brain are key publishers in this field. These results highlight the central role of AI in advancing the diagnosis and prediction of AD and MCI, emphasizing the growing international contribution to this important area of research. We compiled the number of articles published by authors and analyzed the co-citation situation of each author. Shen Dinggang has the highest citation count, publishing 11 articles among the top 100. The author’s research has played a key role in advancing ML technologies for the diagnosis of AD and MCI, with a clear focus on DL, multimodal integration, and early disease detection. In the early stages, Shen’s research applied pattern classification methods to MRI scans to detect prodromal AD in MCI patients, achieving a diagnostic accuracy of up to 90%, and highlighting the diagnostic potential based on MRI (Davatzikos et al., 2008). Around 2011, the research expanded to methods involving more modalities, introducing multi-task learning to predict various clinical variables and AD classification by integrating MRI, FDG-PET, and CSF data (Zhang et al., 2012). Between 2014 and 2017, the author increasingly incorporated DL techniques, such as the Deep Boltzmann Machine (DBM) and Stacked Autoencoder (SAE), to create hierarchical feature representations from neuroimaging data (Suk et al., 2014; Cheng et al., 2015; Li et al., 2015; Suk et al., 2015; Suk et al., 2016). These models could automatically extract high-level features from MRI and PET scans, outperforming traditional hand-crafted feature-based approaches in classification tasks. During the period from 2017 to 2019, the author conducted research on multi-task multi-channel learning for brain disease classification and clinical score prediction using neuroimaging data along with demographic or genetic information, aiming to achieve higher accuracy (Liu et al., 2019; Zhou et al., 2019). Shen’s recent work focused on layered fully convolutional networks to locate brain atrophy in MRI scans, enhancing diagnostic accuracy and tracking disease progression (Lian et al., 2020). We compare two of Shen’s influential studies to reflect the methodological evolution in his research trajectory. Zhang et al. (2012) applied multi-modal multi-task (M3T) learning, integrating MRI, FDG-PET, and CSF features using a multi-task feature selection followed by a multi-modal SVM for regression and classification (Zhang et al., 2012). This approach effectively leveraged complementary information but relied on hand-crafted features and a relatively fixed processing pipeline. In contrast, Zhou et al. (2019) proposed a stage-wise deep neural network, first learning modality-specific latent representations, then progressively combining these features across modalities, and finally fusing them for diagnostic prediction in an end-to-end deep learning framework (Zhou et al., 2019). This evolution from feature-level fusion to hierarchical, data-driven fusion networks marks a significant shift toward more flexible, scalable models capable of better handling multimodal, heterogeneous datasets and incomplete data. The co-occurrence analysis of all keywords revealed emerging research trends and hotspots in this field. Both SVM and CNN are among the top 10 keywords in ML methods. In these studies, the SVM was used the most frequently, appearing in 25 articles. SVM is an ML algorithm primarily used to classify data into multiple predefined categories. By finding an optimal separating hyperplane in a high dimensional space, SVM can classify samples based on feature data; for instance, in neuroimaging research, it can group patients and healthy controls (Gao et al., 2022; Gaonkar et al., 2015; Sacchet et al., 2015). This process includes feature extraction and selection, training and testing classifiers, and evaluating their performance to optimize the algorithm’s predictive capability (Orrù et al., 2012). SVM is particularly well-suited for handling complex classification problems and can efficiently map data using kernel methods, enhancing classification accuracy in high-dimensional data.

A study titled “Using SVM to identify imaging biomarkers of neurological and psychiatric disease: a critical review” (Orrù et al., 2012), which describes the use of SVM to analyze MRI and PET for early diagnosis of MCI and AD, is the most cited article. Of the top 100 cited articles, 19 used SVM to analyze MRI, and 5 analyzed PET. This seems to indicate that the use of SVM methods to analyze patients’ neuroimaging data for early diagnosis of MCI and AD is a major research hotspot. It is worth noting that another paper published in 2020 used SVM methods in the analysis of EEG (Ieracitano et al., 2020). EEG has been found to have the potential to assist in the diagnosis or prediction of a variety of diseases such as AD, perioperative neurocognitive dysfunction (PND), MCI, subjective cognitive decline (SCD), and depression (Boby and Veerasingam, 2025; Ouchani et al., 2021; Deiner et al., 2015; Engedal et al., 2020). Moreover, AI methods facilitate a more convenient and accurate analysis of EEG results (Huang et al., 2012; Hosseini et al., 2021). Utilizing methods such as SVM to analyze patients’ EEG for diagnosing AD or MCI may be a future research direction. Additionally, PET and CSF have long been used to detect molecular and biochemical characteristics related to AD, but their usage frequency is still significantly lower than that of MRI in the current highly cited literature. Sufficient attention should be paid to these relatively understudied modalities and their integration with MRI information is expected to further enhance the sensitivity and specificity of the model for disease progression. For instance, MRI can provide structural or functional changes, while PET and CSF reflect pathological processes such as amyloid and Tau; EEG provides highly time-resolved functional dynamics. The strategy of multimodal integration may become an important approach for promoting the clinical application of AI methods. The frequency of CNN usage is also quite high, with 24 articles employing this method. CNN is a type of DL model particularly suited for processing image data. Compared to traditional methods, CNNs are better at capturing subtle changes in images, thereby enhancing the capability for early diagnosis. In the studies we included, the application of CNN methods for examining AD achieved a sensitivity of 97.96% and a specificity of 97.35% (Wang et al., 2018). A study conducted in 2022 reported a 100% accuracy in classifying AD and Normal Control (NC) subjects using CNN methods (Habuza et al., 2022).

To further contextualize our findings, we compared our bibliometric results with those of a recent comprehensive analysis by Song et al. (2025), which examined 2,316 AI-related AD papers from 2004 to 2023. In line with our findings, both studies identified the United States and China as the leading contributors, but our focus on the top 100 cited articles revealed a more pronounced dominance of the United States (54% of publications vs. 29% in Song et al.), suggesting that the most influential research is concentrated in a few top-tier American institutions such as the University of North Carolina at Chapel Hill and the University of Pennsylvania. In contrast, Song et al. reported more balanced global contributions, with China showing rapid growth but lower citation impact. Journal patterns also differed: our top-cited articles were mainly published in high-impact Q1 journals (e.g., NeuroImage, Brain), whereas Song et al. found higher productivity in broader-scope journals such as Journal of Alzheimer’s Disease and Frontiers in Aging Neuroscience. This implies that high-impact studies tend to cluster in neuroimaging and methodological domains, while the broader field encompasses more clinical and interdisciplinary work. Methodologically, both analyses highlighted DL and ML approaches, yet our dataset showed a greater presence of established algorithms such as SVM and CNN among highly cited works, reflecting their enduring influence and reliability. These comparisons suggest that the top-cited literature represents a distinct high-impact subset characterized by concentration in leading institutions, Q1 journals, and robust AI methodologies, complementing the broader bibliometric patterns of the entire field. In addition, we analyzed the IF, JCR Category Quartile, JCR category, and category of these articles, hoping to help future researchers better submit their papers. Of the 100 articles, 68 were classified within the JCR Q1 zone, which also reflects the importance of this research area. The novelty of our study lies in its exclusive focus on highly influential works, covering both AD and MCI, and its integration of AI methods with clinical data modalities. This multidimensional approach provides insights into the methodological and thematic features that drive high scientific impact in AI-assisted dementia research.

## 5 Limitations

This study is not without its limitations. Despite our endeavors to employ a diverse array of search terms, it is possible that key studies have been overlooked. The ever-evolving nature of citation counts for articles means that recently published papers may not have had adequate time to amass significant citations, thereby excluding them from our analyses. Future updates could incorporate normalized metrics to better capture emerging high-impact studies. Moreover, our article retrieval was not exhaustive across all databases, inevitably leading to some omissions in the collected literature. Additionally, in this study, AI methods are classified into traditional ML and DL categories. However, the delineation is not always clear-cut, as some techniques can straddle multiple categories. For example, SVM, a conventional ML approach, is occasionally incorporated into DL frameworks, blurring the lines of classification. Finally, our analysis summarized AI method frequencies in aggregate but did not classify these methods by publication year, and future work incorporating year-based stratification may help clarify how approaches such as CNN and SVM have evolved over time.

## 6 Conclusion

We analyzed the top 100 cited articles on AI in AD and MCI by examining authors, journals, countries, institutions, keywords, and AI methods. According to these analyses, we find that the application of AI methods to diagnose AD or MCI is the primary research direction among these 100 articles, and MRI data is the most common type of clinical specimen collected for such studies. Moreover, SVM and CNN are the most commonly used AI methods.

## Data Availability

The original contributions presented in this study are included in this article/supplementary material, further inquiries can be directed to the corresponding authors.
